# Communicating uncertainty - how Australian television reported H1N1 risk in 2009: a content analysis

**DOI:** 10.1186/1471-2458-11-181

**Published:** 2011-03-24

**Authors:** Andrea S Fogarty, Kate Holland, Michelle Imison, R Warwick Blood, Simon Chapman, Simon Holding

**Affiliations:** 1 School of Public Health, University of Sydney, Sydney, Australia; 2 Faculty of Arts & Design, University of Canberra, Canberra, Australia

## Abstract

**Background:**

Health officials face particular challenges in communicating with the public about emerging infectious diseases of unknown severity such as the 2009 H1N1(swine 'flu) pandemic (pH1N1). Statements intended to create awareness and convey the seriousness of infectious disease threats can draw accusations of scare-mongering, while officials can be accused of complacency if such statements are not made. In these communication contexts, news journalists, often reliant on official sources to understand issues are pivotal in selecting and emphasising aspects of official discourse deemed sufficiently newsworthy to present to the public. This paper presents a case-study of news communication regarding the emergence of pH1N1.

**Methods:**

We conducted a content analysis of all television news items about pH1N1. We examined news and current affairs items broadcast on 5 free-to-air Sydney television channels between April 25 2009 (the first report) and October 9 (prior to the vaccine release) for statements about [1] the seriousness of the disease [2] how the public could minimise contagion [3] government responses to emerging information.

**Results:**

pH1N1 was the leading health story for eight of 24 weeks and was in the top 5 for 20 weeks. 353 news items were identified, yielding 3086 statements for analysis, with 63.4% related to the seriousness of the situation, 12.9% providing advice for viewers and 23.6% involving assurances from government. Coverage focused on infection/mortality rates, the spread of the virus, the need for public calm, the vulnerability of particular groups, direct and indirect advice for viewers, and government reassurances about effective management.

**Conclusions:**

Overall, the reporting of 2009 pH1N1 in Sydney, Australia was generally non-alarmist, while conveying that pH1N1 was potentially serious. Daily infection rate tallies and commentary on changes in the pandemic alert level were seldom contextualised to assist viewers in understanding personal relevance. Suggestions are made about how future reporting of emerging infectious diseases could be enhanced.

## Background

In recent years, Australians have been exposed to a range of large-scale news coverage and health promotion campaigns about communicable disease. These have included seasonal influenza advisories; campaigns promoting immunisation for vaccine-preventable diseases[1]; traveller vaccination messages; sexually-transmitted disease prevention campaigns[2], including human papilloma virus vaccine to prevent cervical cancer[3]; HIV/AIDS and hepatitis B and C prevention. With the exception of HIV/AIDS and sexually-transmitted diseases, each of these has a vaccine and clear directives about how to avoid infection, forming the central communicative focus of such campaigns.

The WHO-declared global pH1N1 (swine 'flu) pandemic of 2009 has attracted research attention from virologists and infectious disease specialists [4-10], but less from communication scholars [11-14]. From the first reports of Mexican cases in late April 2009, what would become sustained Australian reportage rapidly turned to the likelihood of Australian cases involving perhaps epidemic and high mortality numbers. Australians were exposed to daily news featuring the country's senior health officials and an array of infectious disease experts, who unavoidably, had to deal with the uncertain and complex trajectories and virulence of the disease in the context of news production cultures characterised by seven second sound-bites [15] and an appetite for unambiguous, easily understood information.

For the first five months of the pH1N1 epidemic no vaccine was available. As such, this period represents a prolonged example of news coverage of an uncertain risk with the potential to become a major threat to public health and where medical science had no protection to offer, high-lighting once again the context-specific nature of any risk or crisis communication strategy [16]. Knowledge of the H1N1 virus was characterised by uncertainty about the potential seriousness of the threat and actions that individuals and governments should take. This marked the disease and public news discourse about it as a compelling case study in the high-profile communication of uncertainty.

Prior to pH1N1, Australia had experienced large-scale warning campaigns delivered principally via news reports and government advisories in relation to SARS (November 2002-July 2003)[17] and avian influenza (mainly H5N1) (May 2005 - 2006)[18]. Reportage had much in common with that for H1N1: they were "foreign" in origin; they threatened to arrive on Australia's shores; their endemicity and expected virulence were uncertain; and a prudent suite of behaviours or government actions were not prescribed as ways of minimising infection [19]. Together, these common features - coupled with the failure of these epidemics to materialise in Australia and the failure of the public to develop any significant concern[20] - lent a further degree of interest to pH1N1 and the way in which the government, health authorities and news media tried to communicate risk.

Media risk research highlights how risk is communicated to the public: disseminating risk information; generating and determining public acceptability of different risks; framing responsibility for[21] and motivating action regarding risks; and seeking to explore differences between the communication of voluntary and involuntary risks[22]. Repeated surveys have found around 40% of the public follow health news, with news on influenza being one of the most closely-followed health issues[23]. Studies of the impact of public warnings about emerging diseases with uncertain trajectories have shown that they can attract disproportionate news coverage relative to the burden of disease arising from them [24]. While some studies report that this coverage is often episodic, sensational and contains little information useful to the public in deciding what they should do [25,26], others find that it can be mostly "neutral" and that it can cycle through alarmist reports which tend to calm as more information emerges[11]. Despite perceptions of "excessive" news coverage, personal concern and knowledge of such diseases can remain low [27] but a significant proportion engage in increased infection control measures such as hand-washing [28].

Media-risk research demonstrates that reportage is often governed by journalistic values and organisational norms and has little parallel to actual threat [29-31]. Reporters tend to adopt reporting frames provided by dominant institutions and their key actors who are perceived by journalists as legitimate sources [32]. In the face of hundreds of conflicting findings Bakir (2010) rhetorically asks whether there is anything different in *risk *reportage compared to other types of news. One answer, she says, 'lies in the uncertainty and value judgements inherent in risk issues, and the diverging interpretations they generate' (pg 7). Indeed, as the Editor-in-Chief of the *Journal of Health Communication *put it, the confusion and uncertainty about the pH1N1 threat was 'communicable chaos'[12] (pg 413).

No studies appear to have been published on how emerging diseases are treated by television news. This is a significant absence, because television news on a typical weeknight in Australia can reach over a fifth of the population [33], while the highest state-specific newspaper reaches 1.3 million [34].

## Methods

Since May 2005, the Australian Health News Research Collaboration has recorded and categorised all news, current affairs and 'infotainment' programmes related to health and medicine on Sydney free-to-air television stations[15]. We searched the AHNRC database and included all items tagged with 'H1N1' or 'swine flu' in the period April 25, 2009 (the first mention) until October 9 2009. All stories were video clips which were used for the content analysis reported here.

Using a list of content categories that emerged progressively from the content as the pH1N1 story evolved, two authors (AF and MI) compared coding on a set of 15 random clips that each watched and coded individually. After resolving any coding differences and agreeing upon how particular items should be handled, they coded the remainder of the items. These categories related to statements made regarding [1] the *seriousness *of H1N1, [2] recommended *actions viewers were advised to take *about avoiding contracting or spreading pH1N1, and [3] reassurances that the government was handling the situation. A statement was any direct (X said "Y") or attributed ("X said that...") quote by the journalists or news actors featured in each item. A test of inter-coder reliability produced a Kappa statistic of 0.63, indicating a good level of agreement[35].

## Results

A total of 353 news stories were identified, containing 3,086 statements related to the three key areas of inquiry. During the 24 weeks reported here, pH1N1 was the leading health story for eight weeks and for 20 weeks remained in the top five most frequently reported health stories. We also note that the virus was rarely referred to by the name pH1N1 during the coverage and instead, was routinely termed swine 'flu. When reporting exact quotes we have therefore retained the term swine 'flu.

### 1) Seriousness Of pH1N1

Of all statements, 63.4% (n = 1,958/3,086) related to the seriousness of pH1N1 (Table 1). This was communicated via four recurring stories: (i) daily tallies of infection and mortality; (ii) descriptions of spread of the virus; (iii) the need for calm responses; and (iv) the vulnerability of particular groups. We briefly summarise other statements which did not comprise significant proportions of the coverage, but may have been important to those who incidentally saw some news stories. This included similarities between pH1N1 and other viruses, government management plans, and the need for systems covering diagnosis and the anticipated vaccine roll out.

**Table 1:** Statements concerning the seriousness of H1N1

Statements	N	(%)
Infection and mortality rates	836	42.7

Outbreaks, pandemics, the quick spread of the virus or an inability to contain it	221	11.3

Statements meant to calm any panic and reassure the viewer	151	7.7

Statements concerning particular risk groups (e.g. pregnant women, people with underlying health conditions etc.)	145	7.4

Comparisons with other viruses (e.g. SARS, seasonal flu, 1918 pandemic)	135	6.8

Containment measures (e.g. border control, quarantine, school closures, sporting cancellations)	101	5.1

Government Pandemic Preparedness Plan	86	4.4

Changes to WHO or Australian government alert levels	76	3.9

Vulnerability to H1N1 (e.g. lack of immunity, called 'potentially deadly virus', everybody at risk)	64	3.3

Vaccine (e.g. need for a vaccine, testing and rollout of vaccine)	61	3.1

Testing and diagnosis of H1N1 strain	58	3.0

Other	24	1.2

**TOTAL**	**1,958**	**100.0**

#### (i) Infection and mortality tallies

The most common (42.7%, n = 836/1,958) statements communicating the seriousness of H1N1 concerned infection and mortality rates. These generally conveyed that infection rates were serious (*"Australia is the most severely-affected country in the Asia Pacific with nearly 12,000 cases: more than 10% of the global total"*), universal and spreading (*"Cases have now been confirmed in Mexico, the US, Canada, Spain, the UK, Israel, New Zealand, Germany, Austria and Costa Rica, with cases suspected in South Africa") *and could result in hospitalisation (7.7%, n = 64/836; *"...nearly half of the 123 patients hospitalised around Australia with swine 'flu are in intensive care..."*).

Statements about deaths initially concerned Mexico, but switched to Australia once local deaths occurred among those with underlying medical conditions (*"A second Victorian has died ... She was 50 years old and had cancer..."*). This message changed to encompass people of all ages, as well as risk groups outside those traditionally vulnerable to seasonal 'flu (*"they're young, healthy and at risk of dying from swine 'flu: several hospitals are now reporting cases of otherwise fit people ending up in intensive care..."*). A key concern was accuracy of predictions and the salience given to uncertainty regarding how high these rates might climb (*"It's really impossible to honestly make those predictions. The fear of course in the international community is that it could get very bad, very quickly..."*).

#### (ii) Describing the speed and spread of the virus

Coverage also referred to the spread using descriptive language punctuated by epidemiological terms (11.3%, n = 221/1,958). The WHO declared an official pandemic on June 11, 2009 (*"I have therefore decided to raise the level of influenza pandemic alert from phase 5 to phase 6. The world is now at the start of the 2009 influenza pandemic."*). Statements provided commentary that suggested multiple, rapidly-spreading outbreaks which were difficult to contain *("swine 'flu has been declared unstoppable"*). Descriptions often suggested uncertainty to viewers: for example, some organisations cancelled public events ("*The grand prix swimming meet won't go ahead in Melbourne later this month as sports administrators there and elsewhere take pre-emptive action to stop the virus spreading"*), only to be told by the government this was unnecessary (*"I would urge those who are organising events to make sure that their decisions are based on medical advice"*). Other events went ahead as planned ("*tonight's [football] match ...will go ahead, but without Queensland's State of Origin players, who are in quarantine after one of their number tested positive"*) albeit with some confusion as to whether this was appropriate ("*players say measures to stop the virus spreading are disruptive but necessary" *versus *"some health experts think quarantining the players is an over-reaction.")*

#### (iii) The need for calm responses from the public

Despite acknowledgement of uncertainty, health authorities sought to reassure viewers of the importance of remaining calm (*"I don't think there's any reason for alarm or anxiety in the community ... so I think let's keep perspective on this")*. Among all reassuring statements across the 3 questions (n = 182), 34.1% (n = 62/182) stated that the virus was not as severe as first feared; 20.3% (n = 37/182) were statements from health authorities about their confidence in the pandemic preparedness plan; 14.8% (n = 27/182) were indirect statements that implied the level of fear was disproportionate; and 13.7% (n = 25/182) were direct entreaties for the public to not panic. While some statements are necessarily time dependent, collectively they nevertheless reflect the continuous nature of the reassuring messages present throughout the sample period.

#### (iv) Vulnerability of particular groups

Several statements implied only particular groups were vulnerable (7.4%, n = 145/1,958), while others implied that everyone was at risk (3.3%, n = 64/1,958). Those with underlying health conditions were considered particularly at risk and in need of anti-viral medication (*"...the 'flu can have severe outcomes for some people with existing conditions ... those conditions include morbid obesity, cardiovascular, renal and respiratory diseases")*, as were pregnant women (*"..the sharp increase in the number of pregnant women in intensive care, six are now on life support with swine flu, most have had their babies delivered prematurely..."*). Yet other statements implied that infection did not match previous patterns, making everybody vulnerable to poor outcomes (*"...because of a lack of immunity in the community we are seeing more serious disease in people that were previously well...")*.

It was therefore unclear whether everyone was vulnerable to infection and poor outcomes, including those who were well and not part of the usual high-risk groups, or whether everyone was vulnerable to infection but only some groups vulnerable to poor outcomes. However, as time went on and more became known about the illness, health authority messages sought to clarify uncertainty *("the important thing is to say that mostly people have the normal experience with the 'flu and will be fine... What we're looking for is people with underlying medical conditions that suddenly get worse, or ordinary people that've not got other conditions that start to deteriorate").*

#### (v) Other information

The seriousness of pH1N1 was also communicated via comparisons with other viruses such as SARS, avian and seasonal 'flu. Yet there was disagreement as to whether pH1N1 was less severe (*"...we've got 148 confirmed cases of pH1N1 globally, we have over 8.5 thousand seasonal influenza, human influenza every winter. We need to put this in perspective..."*), more severe (*"infectious diseases experts say the threat is worse than SARS or the bird 'flu..."*) or similar to other familiar viruses (*"...the next few days will show whether this is just another 'flu strain that happens to have swine and avian elements, or whether indeed it is a significant killer 'flu..."*).

### 2) Advice And Recommended Actions For Viewers

In more than one third of stories (n = 131/353 - 37%) direct or indirect advice was given on what viewers could do to prevent spreading infection (table 2). However, these statements accounted for just 12.9% (n = 399/3,086) of all statements. Just over a quarter (27.8%, n = 111/399) focused on basic personal hygiene, another quarter related to preventing infection by being mindful of issues of proximity (27.8%, n = 111/399) and a fifth advised seeing a doctor and seeking further information (20.6%, n = 82/399)..

**Table 2:** direct and indirect advice to viewers

Statements	N	(%)
Look after basic hygiene (e.g. wash hands, cover your mouth when you cough etc)	111	27.8

Help prevent the spread of infection (e.g. stay home if you are sick, avoid crowds, defer non-essential travel, respect quarantine, keep sick children home etc.)	111	27.8

Seek credible information, see a doctor and/or take medication as advised	82	20.6

Consider a vaccine*	38	9.5

Be alert if you belong a particular risk group	35	8.8

Other	22	5.6

**TOTAL**	399	100.0

*Note: the H1N1 vaccine became more prominent in coverage occurring outside the time period reported here

#### Conflicting advice

Of all statements (n = 399) concerning advice on what to do just 3.5% provided conflicting or unclear information. This could involve government health authorities disagreeing (*"I think it's prudent that all households think about their readiness...." *versus *"there is no need at this point for people to start thinking about those preparations..." *in the same bulletin), or unclear advice concerning face-masks (e.g. *"pregnant women are being advised to wear face-masks in public..." *versus *"we don't all need to be donning masks. The people that should be wearing masks, whether it's swine 'flu, seasonal influenza or any kind of cold virus, are the people that have the symptoms..."*) yet many clips featured imagery of the public wearing masks, while not being pregnant or apparently symptomatic. Likewise, people were advised to respect quarantine measures, but some news actors did not model this behaviour (*"Senator Fielding has defied calls to stay at home despite having potentially been exposed to swine 'flu..." *and "*swine flu victim Karmichael Hunt will play for the Broncos against the Bulldogs tonight in Brisbane"*). Yet the uncertainty about what to do was acknowledged by commentators ("*they are doing to a large extent all they can, they're seeking medical advice, which is often conflicting unfortunately, and following the advice of the experts and that's really all they can do, shy of shutting down an entire sport"*). These conflicting statements were, however, a very small proportion of the advice offered to viewers.

### 3) Reassurance That Government Was Handling The Situation

Of all statements recorded, 23.6% (n = 729/3,086) assured viewers that the government was handling the situation by elaborating on its current and proposed actions (Table 3).

**Table 3:** Reassurance through government action

Statements	n	(%)
Vaccine (e.g. production, testing and roll-out; advice about priority groups etc)	217	29.8

Border control and containment measures (e.g. quarantine, thermal imagery, health declaration cards)	189	25.9

Government pandemic preparedness plan and alert level	126	17.3

Administration of the anti-viral stockpile	45	6.2

Closures of public areas and postponement of public events	42	5.8

Testing and diagnosis of the H1N1 strain	38	5.2

Advise the public to remain calm	26	3.6

Identification of risk-groups	22	3.0

Other	24	3.3

**TOTAL**	729	100.0

About a third of these statements (29.8%, n = 217/729) referred to the immediate need for the Government to develop, test and then distribute a vaccine starting with priority groups (*"we will purchase ... doses to cover 10 million people based on the current expert advice that this is sufficient to contain the spread of the disease"*).

A quarter of these statements (25.9%, n = 189/729) reassured the public that the government was putting significant effort into border control measures designed to prevent pH1N1 entering Australia, and following up and containing detected infection. These statements generally concerned quarantine measures (*"extra quarantine measures were today instituted for passengers arriving from overseas, with medical staff deployed at airports..."*), the use of thermal imaging at airports or statements about new measures and ongoing monitoring of the situation (*"...the Governor-General has signed off on sweeping new detention and surveillance powers in case the authorities need to act quickly..."*).

Many statements concerned the official pandemic preparedness plan [36] (17.3%, n = 126/729) and the Government's willingness to take action ("*this is a serious matter, the government takes it seriously, all necessary resources will be deployed to meet the threat, calibrated to how it unfolds"*), assurances about communication between the government and health authorities (*"we've taken [the Chief Medical Officer's] advice and we will continue to take his advice..."*) and statements that the plan was an effective tool and that the required services were on standby (*"we have a very well-developed plan between the states and territories ...we've got a very strong health system, our GPs and emergency departments are well-informed with how to deal with presentations if the matter becomes more serious... "*).

These assurances were accompanied by acknowledgment of uncertainty, including the handling of a cruise ship with infected crew on board (*"more than a thousand passengers are in lock-down tonight, stuck on a cruise liner in Sydney harbour because of a swine 'flu scare") *and the ensuing confusion when some passengers were allowed to disembark and others weren't ("*I think the response was complicated by both the Commonwealth and New South Wales, and I think that in the future we need to learn to integrate our response..").*

#### News actors

Of all statements, the majority were made by reporters (53.6, n = 1,653/3,086); a further 21.5% (n = 662/3,086) by representatives of the government, including their medical officers; and 12.2% (n = 376/3,086) by public health and infectious disease experts. The remaining 12.8% (n = 395/3,086) were a combination of *vox populi *statements and comments from overseas officials, athletes or other stakeholders.

## Discussion

pH1N1 sustained large-scale news coverage over many months, a media focus commensurate with the seriousness with which major health organisations and governments approached the disease. Despite the uncertainty surrounding the virus, statements made about pH1N1 were generally non-alarmist and reassuring, in keeping with findings related to newspaper coverage[37]. As with all studies focused on the content of news texts alone, a limitation of this study is that we are unable to draw any conclusions about the impact of coverage on audiences. In using statements as the unit of analysis we also acknowledge that conclusions cannot be drawn about individual news stories in context. However, we do find areas for consideration regarding the messages potentially taken away by news consumers.

The emerging pandemic saw reportage fuelled by the daily release by the WHO and Australian government of data on new infections and deaths[38,39]. The volume, duration and much of the content of the coverage was such that the public could hardly avoid concluding that the disease was serious enough to warrant the prominent attention it was receiving. The initial sub-text was that pH1N1 might have been shaping to be "the big one": a pandemic that could threaten many lives. While Australia and the world saw relatively few deaths, the possibility that this might change underscored the enduring media attention and government vigilance. This, combined with the audience's experience of other prominent health campaigns in recent years, may have created audience expectations that reportage would include information of a less "banal" nature on important practical strategies for risk reduction.

Health officials openly acknowledged the uncertainty regarding how the pandemic might unfold, yet such statements were accompanied by authoritative reassurance that high-level preparedness existed if worst-case scenarios eventuated: 17% of the government's reassuring statements confirmed they had an effective plan, a stockpile of medication and the will to take action as necessary. Viewers were often reminded to seek further advice by visiting the government website, calling the information line or contacting a doctor.

But beyond advice that the public should remain alert and keep informed, and that the government was well prepared, few clear directives were given on what viewers should actually *do *themselves. Routine advice that mirrored advice for reducing contagion from common colds and 'flu was given regarding basic hygiene (e.g. hand-washing, covering mouth when coughing, etc) and containing the spread of infection (e.g. staying at home with 'flu-like symptoms, deferring non-essential travel, adhering to quarantine associated with school closures, etc.) - but overall this accounted for only 13% of all statements.

This low key and relatively infrequent advice may have imbued viewers with a sense that pH1N1 was nothing out of the ordinary and helped prevent undue alarm arising. Yet occasional news incidents which belied any sense of banal risk may have fomented some dissonance in viewers. While such items did not make up the bulk of the reportage, they risked creating alarm in viewers who may have only seen occasional news stories. Early in the reporting period, a cruise ship with infected passengers and crew was reported over several days, with undertones that it was a potential conduit for the virus to spread into different Australian cities. Items containing contradictory risk information sometimes were broadcast within the same news bulletin (e.g. a lead news item that the WHO had declared a pandemic, adjacent to a sports news story about a recently infected footballer who had been cleared to play; footballers together in a spa adjacent to a story about the cancellation of a national elite swimming event; Government ministers talking about increased airport measures, with *vox populi *of disembarking passengers saying they had experienced no border control screening etc.). These inconsistencies had the potential to undermine the government's assurances that everything was under control but they may also have encouraged viewers to seek out more information about the disease.

Other than the inconsistencies described - a product of different reactions and responses to the risk posed by pH1N1 in the community - we saw little to no evidence of editorial effort to sensationalise reportage by, for example, highlighting examples of community anxiety or the views of any "maverick" experts arguing that the assurances being given were irresponsible or, conversely, that the seriousness with which the threat was being taken and the public health measures put in place had been exaggerated. For the most part, reports were moderate or simply updating previous news.

It is also important to recognise the limitations of accusing the media of sensationalism or lack of balance in that it risks obscuring rather than clarifying the processes that influence media coverage. For example, as Kitiznger notes [40]determining what constitutes alarming or reassuring statements or media coverage is no simple task and is very much dependent on the situation in question as to whether one believes people *should *be alarmed or reassured.

We recognise the time constraints facing television news journalists as well as the different expectations that viewers bring and the factors that shape their interpretations of risk. Notwithstanding these factors our study identifies opportunities where reporting of emerging diseases could be enhanced. While there were daily tallies of infection rates with commentary on how this related to changes in the pandemic alert level, there was little apparent effort to contextualise what the figures meant. Viewers might hear that pH1N1 had arrived in Australia, yet still have little understanding of their personal vulnerability or how the number of infections and deaths compared with those from other infectious diseases like seasonal 'flu or whooping cough, which was also affecting the community at the time [41]. We suggest that constant updates to infection rates could be improved by providing commentary that either (a) would allow a viewer to reach conclusions about their own level of risk or (b) reiterated useful advice about reducing infection and contagion at home and in the community at large, especially given that in other countries viewers felt infection was inevitable and not easily preventable[42].

News coverage of pH1N1 as an uncertain and potentially serious risk raised serious challenges for risk communication. With both SARS and avian 'flu having recently attracted mass reportage yet not causing any deaths in Australia, potential "cry wolf" [43] legitimacy risks faced Australian health authorities. Concerns over the catastrophic potential of foreign-origin infections had proved unwarranted twice before, so further major public alerts with similar outcomes might engender a sense of either cynicism or complacency. On the other hand, authorities risked the charge that they had not done enough in the event that the disease caused many deaths. These competing concerns created a challenging communication context for health authorities and journalists alike.

It would have been unacceptable for the government to promote personal risk reduction practices like working from home, avoiding crowds and discretionary travel, and the wearing of facemasks if such measures were unwarranted by the risk assessments available. However, it may have been sensible to advise the community of what situation would need to occur before such measures would be recommended. Such advice may have gone some way to promoting greater community understanding of the phases of pandemic preparedness.

There was also the difficulty of communicating complex information within the narrow time constraints of television news, something further compounded by urging viewers to remain calm while simultaneously acknowledging that authorities did not have answers to several questions. Given that a vaccine was not available throughout this sustained period of uncertain risk communication and that mortality remained low, the potential for a 'cry wolf' affect may have been magnified.

Judging by the frequency of press releases across the sample period and the frequency with which the health authorities were available to the media, decisions were plainly taken that the public should be maximally informed, despite there being little different to say from day-to-day in terms of advisories. Almost-daily efforts to generate news coverage were framed by the media in terms of updates and developments, mainly focussed on case increases. For the news media's part, the commercial imperatives to produce compelling news items to attract large audiences for their advertisers may have been tempered by the government's concern to underline the potential seriousness of the disease and journalistic notions of socially responsible reportage. The frequency and volume of news coverage that pH1N1 attracted was, arguably, not reflective of its impact in the community, and understanding the implications of this is an area that warrants further investigation.

## Conclusions

The Australian government and media acted responsibly by providing regular, high profile and highly transparent information on the emerging intelligence about the pandemic. Uncertainty about the trajectory of the disease was openly acknowledged and reassurances given about government preparedness. Potentially dissonance-generating news coverage that might have engendered panic, complacency or cynicism about "yet another epidemic" was uncommon. Opportunities were lost to inform the public about possible future developments and the personal risk reduction behaviours that might then be recommended or mandated.

Further research should explore [1] how various publics decoded and received the information and advice provided and [2] how journalists approached the challenges of making this on-going story maximally newsworthy. Triangulated with content analysis of what was broadcast, such as this paper, these studies could provide valuable information for risk communicators in areas characterised by the uncertainty inherent in emerging diseases.

## Competing interests

The authors declare that they have no competing interests.

## Authors' contributions

AF participated in study conception and design, analysed half of all data and helped draft the manuscript. KH participated in study design, advised on risk communication research and helped draft the manuscript. MI participated in study conception and design, analysed half of all data and helped draft the manuscript. RWB participated in study design, coordination and helped draft the manuscript. SC conceived of the study, coordinated the analysis and helped draft the manuscript. SH provided preliminary coding of all news clips and helped identify relevant news items.

All authors read and approved the final manuscript.

## Author information

All authors are members of the Australian Health News Research Collaboration and invite collaboration with any health researchers interested in news media reporting of health problems. For details see here: http://sydney.edu.au/medicine/public-health/AHNRC/

## Pre-publication history

The pre-publication history for this paper can be accessed here:

http://www.biomedcentral.com/1471-2458/11/181/prepub

## References

[B1] Department of Health and Ageing Immunise Australia Program 2010 Canberra: Department of Health and Ageing

[B2] Department of Health and Ageing Think STIs don't happen to people like you? 2010 Canberra: Department of Health and Ageing

[B3] Department of Health and Ageing Human Papillomavirus (HPV) 2010 Canberra: Department of Health and Ageing

[B4] Webb SA Pettila V Seppelt I Bellomo R Bailey M Cooper DJ Critical care services and 2009 H1N1 influenza in Australia and New Zealand N Engl J Med 2009 361 20 1925 34 10.1056/NEJMoa0908481 19815860

[B5] Depoortere E Mantero J Lenglet A Kreidl P Coulombier D Influenza A(H1N1)v in the southern hemisphere--lessons to learn for Europe? Euro Surveill 2009 14 24 19555604 10.2807/ese.14.24.19246-en

[B6] Anikeeva O Braunack-Mayer AJ Street JM How will Australian general practitioners respond to an influenza pandemic? A qualitative study of ethical values Med J Aust 2008 189 3 148 50 18673101 10.5694/j.1326-5377.2008.tb01948.x

[B7] Bishop JF Murnane MP Owen R Australia's winter with the 2009 pandemic influenza A (H1N1) virus N Engl J Med 2009 361 27 2591 4 10.1056/NEJMp0910445 19940287

[B8] Cheng AC Dwyer DE Kotsimbos AT Starr M Korman TM Buttery JP Summary of the Australasian Society for Infectious Diseases and the Thoracic Society of Australia and New Zealand guidelines: treatment and prevention of H1N1 influenza 09 (human swine influenza) with antiviral agents Med J Aust 2009 191 3 142 5 19645641 10.5694/j.1326-5377.2009.tb02722.x

[B9] Kelly HA Grant KA Williams S Fielding J Smith D Epidemiological characteristics of pandemic influenza H1N1 2009 and seasonal influenza infection Med J Aust 2009 191 3 146 9 19645642 10.5694/j.1326-5377.2009.tb02723.x

[B10] Stuart RL Cheng AC Marshall CL Ferguson JK ASID (HICSIG) position statement: infection control guidelines for patients with influenza-like illnesses, including pandemic (H1N1) influenza 2009, in Australian health care facilities Med J Aust 2009 191 8 454 8 19835543 10.5694/j.1326-5377.2009.tb02886.x

[B11] Duncan B How the media reported the first days of the pandemic (H1N1) 2009: results of EU-wide media analysis Euro Surveill 2009 14 30 19286 19643058 10.2807/ese.14.30.19286-en

[B12] Ratzan SC Swine conflusion J Health Commun 2009 14 5 413 4 10.1080/10810730903034123 19657921

[B13] Seale H McLaws ML Heywood AE Ward KF Lowbridge CP Van D The community's attitude towards swine flu and pandemic influenza Med J Aust 2009 191 5 267 9 19740048 10.5694/j.1326-5377.2009.tb02781.x

[B14] Nerlich B Halliday C Avian flu: the creation of expectations in the interplay between science and the media Sociology of Health & Illness 2007 29 1 46 65 10.1111/j.1467-9566.2007.00517.x 17286705

[B15] Chapman S Holding SJ Ellerm J Heenan RC Fogarty AS Imison M The content and structure of Australian television reportage on health and medicine, 2005-2009: parameters to guide health workers Med J Aust 2009 191 11-12 620 4 20028286 10.5694/j.1326-5377.2009.tb03354.x

[B16] Andersen P Spitzberg B Heath R, O'Hair HD Myths and maxims of risk and crisis communication Handbook of Risk and Crisis Communication 2009 London: Routledge 205 26

[B17] Department of Health and Ageing. Severe Acute Respiratory Syndrome (SARS) 2006 Canberra: Department of Health and Ageing

[B18] Department of Health & Ageing Avian Influenza (Bird Flu) 2008

[B19] Stephenson N Jamieson M Securitising health: Australian newspaper coverage of pandemic influenza Sociol Health Illn 2009 31 4 525 39 10.1111/j.1467-9566.2009.01162.x 19397761

[B20] Seale H McLaws ML Heywood AE Ward KF Lowbridge CP Van D The community's attitude towards swine flu and pandemic influenza Medical Journal of Australia 2009 191 5 267 9 19740048 10.5694/j.1326-5377.2009.tb02781.x

[B21] Iyengar S Is anyone responsible?:how television frames political issues 1991 Chicago: University of Chicago Press

[B22] Bakir V Media and risk: old and new research directions J Risk Res 2010 13 1 5 18 10.1080/13669870903135953

[B23] Brodie M Hamel EC Altman DE Blendon RJ Benson JM Health news and the American public, 1996-2002 J Health Polit Polic 2003 28 5 927 50 10.1215/03616878-28-5-927 14604217

[B24] Berry TR Wharf-Higgins J Naylor PJ SARS wars: an examination of the quantity and construction of health information in the news media Health Commun 2007 21 1 35 44 17461750 10.1080/10410230701283322

[B25] Dudo AD Dahlstrom MF Brossard D Reporting a potential pandemic - A risk-related assessment of avian influenza coverage in US newspapers Sci Commun 2007 28 4 429 54 10.1177/1075547007302211

[B26] Wilson N Thomson G Mansoor O Print media response to SARS in New Zealand Emerg Infect Dis 2004 10 8 1461 4 15496249 10.3201/eid1008.031096 PMC3320422

[B27] Bergeron SL Sanchez AL Media effects on students during SARS outbreak Emerg Infect Dis 2005 11 5 732 4 15890131 10.3201/eid1105.040512 PMC3320361

[B28] Blendon RJ Benson JM DesRoches CM Raleigh E Taylor-Clark K The public's response to severe acute respiratory syndrome in Toronto and the United States Clin Infect Dis 2004 38 7 925 31 10.1086/382355 15034821

[B29] Mythen G Ulrich Beck: a critical introduction to the risk society 2004 London; Sterling, Va.: Pluto Press

[B30] Kitzinger J Researching risk and the media Health, Risk & Society 1999 1 1 55 69

[B31] Murdoch G Kitzinger J Hughes E Taylor-Gooby P, Zinn J The media and risk Risk in social science 2006 Oxford; New York Oxford University Press 250 70

[B32] Kitzinger J Henderson L Smart A Eldridge J Media coverage of the ethical and social implications of human genetic research 2002 Cardiff: The Wellcome Trust

[B33] Knox D TV Tonight Ratings, Week 46 2010 http://www.tvtonight.com.au/2010/11/week-46-3.html [accessed on 22nd November 2010]

[B34] Roy Morgan Research 2010 Roy Morgan readership estimates for Australia for the 12 months to June 2010 http://www.roymorgan.com/news/press-releases/2010/1145/[accessed on 22nd November 2010]

[B35] Fleiss JL Statistical methods for rates and proportions 1981 New York: John Wiley & Sons

[B36] Council of Australian Governments Working Group on Australian Influenza Pandemic Prevention and Preparedness. National Action Plan for Human Influenza Pandemic 2009 Canberra: The Department of Prime Minister and Cabinet

[B37] Holland K Blood RW Not just another flu? The framing of swine flu in the Australian press 2010 ANZCA 2010: Media, democracy and change conference proceedings Canberra, Australia

[B38] World Health Organisation Pandemic (H1N1) 2009 - update 57 2009 Geneva

[B39] Department of Health and Ageing National tally of confirmed cases of H1N1 Influenza 09 2009 Canberra: Department of Health and Ageing

[B40] Kitzinger J Researching risk and the media Health, Risk and Society 1999 1 1 55 69 10.1080/13698579908407007

[B41] AAP Whooping cough cases surge in NSW 2009 http://www.smh.com.au/national/whooping-cough-cases-spread-far-and-wide-20090704-d8e3.html [accessed on 26 November 2010]

[B42] Hilton S Smith E Public views of the UK media and government reaction to the 2009 swine flu pandemic BMC Public Health 2010 10 1 697 PMID: 21078169 10.1186/1471-2458-10-697 21078169 PMC2998491

[B43] Breznitz S Cry wolf: the psychology of false alarms 1984 Hillsdale, N.J.: Lawrence Erlbaum Associates

